# *Helicobacter Pylori* Infection and Lung Cancer: New Insights and Future Challenges

**DOI:** 10.3779/j.issn.1009-3419.2018.09.03

**Published:** 2018-09-20

**Authors:** Ileana GONZÁLEZ, Paulina ARAYA, Armando ROJAS

**Affiliations:** Biomedical Research Laboratories, Medicine Faculty, Catholic University of Maule, Talca, Chile

**Keywords:** *Helicobacter pylori*, Lung neoplasms, Air pollution, Pathogen-associated molecular patterns, Damage-associated molecular patterns, Chronic inflammation

## Abstract

*Helicobacter pylori* (*H*. *pylori*) is the causative agent of chronic gastritis and peptic ulcer diseases and is an important risk factor for the development functional dyspepsia, peptic ulceration, gastric adenocarcinoma and mucosa-associated lymphoid tissue lymphoma. *H*. *pylori* has very high rates of infection in human populations, and it is estimated that over 50% of the world population is infected. Recently, certain extra-gastric manifestations, linked to *H*. *pylori* infection, have been widely investigated. Noteworthy, a growing body of evidences supports an association between *H*. *pylori* infection with lung cancer. The present review intend to highlight not only the most recent evidences supporting this association, but also some missed points, which must be considered to validate this emerging association.

## *Helicobacter pylori* infection

*Helicobacter pylori* (*H*. *pylori*) is a Gram-negative spiral-shaped bacterium that persistently colonizes the human stomach. In this sense, colonization constitutes an established and major risk factor in the pathogenesis of functional dyspepsia, peptic ulceration, gastric adenocarcinoma and mucosa-associated lymphoid tissue lymphoma^[1]^. In fact, and since 1994 *H*. *pylori* has been classified as a Group 1 carcinogen the International Agency for Research on Cancer (IARC)^[2]^.

*H*. *pylori* chronically infects more than half of the world's population, being estimated that this gram-negative bacterium has co-evolved with its human host since its migration out of Africa along with human host approximately 60, 000 years ago^[3]^.

The pathogenesis of *H*. *pylori* mainly depends on the exposure of several bacterial factors to the host, including cytotoxin-associated gene A (CagA), vacuolating cytotoxin A (VacA), type Ⅳ secretion system (T4SS), outer inflammatory protein A, as well as different adherence factors. Because of their critical roles in *H*. *pylori*-induced pathogenesis, these pathogenicity factors have been extensively studied not only in gastric cancer high-risk *H*. *pylori*-infected populations but also on their carcinogenic mechanisms. Although the vast majority of *H*. *pylori* in colonized hosts are free-living, and approximately 20% bind to gastric epithelial cells and adherence is required for prolonged persistence in the stomach and for induction of injury^[4-6]^.

More recently, the scope of the pathogenic mechanisms linked to the oncogenic potential of *H*. *pylori* has been extended to the capacity of this pathogen to produce genomic instability on gastric epithelial cells^[7]^.

Strikingly, gene products from the *cag* pathogenic island (PAI) appear to play an important role in the accumulation of DNA double-strand breaks (DSBs) in infected host cells and the expression of RAD51 is reduced after infection with *cag*-positive strains^[8]^.

Indeed, RAD51 plays an important role in homologous strand exchange, a key step in DNA repair of double strand breaks through homologous recombination (HR)^[9]^.

Additionally, the interactions of *H*. *pylori* with host cells result initially to the adhesion to epithelium, which in turn induce a marked inflammatory responses resulting in persistent colonization, chronic inflammation and severe inflammation, the disruption of the epithelial barrier function^[10-12]^.

Furthermore, the *H*. *pylori* associated gastric cancer is characterized by a chronic inflammatory phenotype, where the long-lasting activation of the key inflammatory regulator nuclear factor kappa B (NF-κB) is essential in contributing to neoplastic transformation^[13, 14]^. At the cellular level, myeloid and lymphocytic cells frequently infiltrate malignant lesions.

Tumor-associated macrophages (TAM) promote malignant progression and the degree of TAMs infiltration correlates with tumor progression and clinical disease stage^[15]^.

## Extra-digestive diseases and *H*. *pylori* infection

During the late 1990s the first reports showing that *H*. *pylori* is associated with extra-digestive diseases appeared in scientific literature^[16-20]^.

Since then, a huge body of evidences has support the association of *H*. *pylori* infection with many extra-digestive diseases including autoimmune diseases, cardiovascular diseases, colonic and pancreatic diseases, diabetes mellitus, hepatobiliary system diseases, neurological disorders, skin diseases, hematological diseases and as well as reproductive disorders^[21-23]^.

The immune and inflammatory responses triggered by *H*. *pylori* infection is postulated as the main mechanisms supporting the extra-digestive pathologies^[24]^.

Of note, recent studies of seroprevalence of *H*. *pylori* in patients, as well as some *meta*-analysis studies have also suggested a significant association between *H*. *pylori* infection and chronic respiratory diseases^[25-29]^. However the pathogenetic mechanism(s) for the effects of *H*. *pylori* in lungs remains mainly elusive.

## The case of lung cancer

Lung cancer remains not only as the leading type of cancer worldwide, but also as as the most common cause of death from cancer^[30]^. Primary lung cancer is known to be caused by either voluntary or involuntary inhalation of environmental carcinogens and where lung cancer cases attributable to smoking has reached up to 90% in countries with a history of tobacco consumption, as reported by IARC^[31]^.

However, 10%-15% of lung cancer cases diagnosed in Western countries and approximately one of every four cases in Asia occur in never smoker subjects^[32]^.

Infectious agents and their potential roles in carcinogenesis have been extensively investigated for many years. In fact, it is estimated that almost 25% of all cancers are somehow associated with chronic infection and inflammation.

Accordingly, several evidences derived from both, epidemiological studies and basic research, have shown that organ-specific carcinogenesis is linked to the development of a chronic and local inflammatory milieu raised as a consequence of host response to infection, as demonstrated for *H*. *pylori* infection and gastric cancer, *Salmonella typhi* and gallbladder carcinoma, human papilloma virus with both oropharyngeal and cervical cancers, and nasopharyngeal carcinoma and Hodgkin's lymphoma with Epstein-Bar virus, just to mention a few^[33-37]^.

In this context, and on spite that some published studies are controversial, the most recent reports support the association between *H*. *pylori* infection and lung cancer^[38]^.

Noteworthy, some recent studies have shed light onto the pathogenic mechanisms involved in this association. Very interestingly, the presence of DNA from *H*. *pylori* detected by Real-time PCR and even some pathogen-derived proteins such as Vac-A, have been found in bronchoalveolar lavage from lung cancer and in lung biopsies specimens, respectively. Additionally, VacA is able to induce induces both IL-6 and IL-8 production in the lung carcinoma cell line A549, as well as Il-8 in human bronchial epithelial cells, just supporting the idea de lung epithelium is responsive to pathogenic factors of *H*. *pylori*^[39, 40]^.

To understand how these bacterial components can be found on lung tissue it is important to highlight that available clinical and experimental evidence points to a possible relationship between the progression of airways disease, pro-inflammatory processes and gastric aspiration^[41, 42]^, and where the main cause is thought to be due tracheobronchial aspiration of small amounts of stomach-associated components, such as pepsin or bile acids, and thus causing repetitive subclinical injury to the lung^[43, 44]^.

Additionally, oral cavity has been suggested as an extra-gastric reservoir of *H*. *pylori*, and thus this pathogen can reach the lungs from either stomach or oral cavity^[45-47]^.

In the case that either *H*. *pylori* or some of its components reach the pulmonary epithelium, it would trigger without any doubt an inflammatory response.

The epithelial lining of the lung abundantly expresses pathogen-recognition receptors (PRRs), like Toll-like receptors (TLRs) to detect a myriad of pathogen-associated molecular patterns (PAMPS). Either lung epithelial cells, alveolar macrophages or dendritic cells, which constitute the first line of lung defense, express TLRs on their surfaces able to recognize not only bacterial-associated PAMPS but also cell-wall and membrane components such as peptidoglycan, lipoproteins, lipoteichoic acid or even pathogen-secreted toxins^[48]^. Furhermore pathogen DNA is recognized by cytoplasmatic surveillance receptors such as TLR-9.

In this context, the receptor of advanced glycation end-products (RAGE) is now recognized as a pathogen-recognition receptor^[49]^. This receptor is abundantly expressed at type-Ⅰ alveolar epithelial cells (AT1), which comprise only 4% of the alveolar surface area, yet they constitute 60% of alveolar epithelial cells and 10%-15% of all lung cells. AT1 cells are large squamous cells that cover 95% of the alveolar surface area and form the epithelial component of the thin air-blood barrier^[50]^. Additionally, RAGE is also expressed in differentiating alveolar type-Ⅱ epithelial cells (AT2), bronchial smooth muscle cells, vascular endothelial cells, and pulmonary macrophages^[51]^.

Noteworthy, TLR-2, TLR-4 and RAGE are involved in the recognition of PAMPS in *H*. *pylori* and thus triggering robust inflammatory response, not only at gastric epithelium^[52]^ but also in monocytes/macrophages^[53]^, dendritic cells^[54]^ and B cells^[55]^.

Interestingly, both TLRs and RAGE can response either directly, by recognizing PAMPs, but also indirectly through the recognition of damage-associated molecular patterns (DAMPs), also known as alarmins, generated as a consequence of cellular stress, damage or cell death, and where the release of DAMPs as a consequence of lung injury has been extensively reported^[56-60]^.

Furhermore, epithelial cell-derived expression of inflammatory mediators after of PAMPs or DAMPs recognition by PPRs, markedly influences the recruitment and activation of immune cells responses in the lungs^[61]^.

Therefore, the chronic exposure to pathogens or pathogens-derived components, oxidants and toxic pollutants causes the release of DAMPs that activate epithelial cell-intrinsic pattern-recognition pathways and also recruit and activate cells of the immune system.

The contribution of air of pollution/smoking deserves a special attention. Of note, it is estimated that there are nearly 1 billion smokers worldwide and approximately 80% live in either low- or middle-income countries where the effects or burdens of tobacco-related illness and death, such as lung cancer, are the most documented^[62]^.

In addition, the World Health Organization (WHO) has estimated that 91% of the world's population lives in places where air quality levels exceed WHO limits, being the low- and middle-income countries those that experience the highest burden^[63]^. On the other hand, about 50% of the world population is infected by *H*. *pylori* and the rate of infections varies from 15.5% for high-incomes countries up to 93.6% for in low- and middle-incomes countries^[64]^.

Therefore, it is likely that a high number of *H*. *pylori*-infected subjects living in low and middle-incomes countries are also exposed to high levels of air pollutants. In this context, it is tempting to speculate, that chronic and subclinical tracheobronchial aspiration in *H*. *pylori*-infected subjects, together with the burden of smoking or air-pollution can act synergically to establish and perpetuate an inflammatory reaction at epithelial lining of the lung, and thus favoring malignant transformation and tumor growth.

Based on the most recent reports suggesting the association between *H*. *pylori* infection and chronic respiratory diseases, and particularly lung cancer, further studies are imperative not only to validate the association, but also to understand the contribution of other factors such as smoking habits or air pollution, and where the underlying molecular mechanisms still remains to be clarified.
